# One‐stage minced cartilage autograft with platelet‐rich plasma improves early clinical outcomes: A multicentric retrospective study

**DOI:** 10.1002/jeo2.70162

**Published:** 2025-02-10

**Authors:** Arthur Barbaret, Frank Wein, Christophe Jacquet, Matthieu Ollivier

**Affiliations:** ^1^ Department of Orthopedic Surgery and Traumatology, St Marguerite Hospital Institute of Movement and Locomotion Marseille France; ^2^ Clinique Louis Pasteur Essey‐lès‐Nancy France

**Keywords:** cartilage, cartilage defect, knee, minced cartilage autograft, PRP

## Abstract

**Purpose:**

Knee cartilage defects are a therapeutic challenge, often requiring multiple costly procedures with modest improvements. This study evaluates whether a one‐stage minced cartilage autograft with platelet‐rich plasma (PRP) improves clinical and radiological outcomes after at least 1 year.

**Methods:**

A multicentric, non‐randomized, retrospective analysis was conducted using data from two sports medicine centres. Sixty‐six patients aged 18–50 years with symptomatic International Cartilage Repair Society Grade III/IV chondral defects underwent the minced cartilage autograft and PRP procedure between January 2021 and April 2023. The minimum follow‐up was 1 year. Clinical scores for Knee Injury and Osteoarthritis Outcome Score (KOOS), International Knee Documentation Committee (IKDC) Score and Self Knee Value (SKV) were collected from medical records and by an independent examiner at the latest follow‐up. Magnetic resonance imaging (MRI) assessments using the Magnetic Resonance Observation of Cartilage Repair Tissue (MOCART) 2.0 scoring system were performed at the latest follow‐up in 46 patients (70%).

**Results:**

The mean follow‐up was 20.5 ± 7.5 months. The study demonstrated significant improvements in knee function and pain relief following the minced cartilage autograft with the PRP procedure. The mean KOOS total score started from 45.3 to 71.5 points, mean KOOS pain started from 51.9 to 80.4 points, mean KOOS activities of daily living started from 62.1 to 85.3 points, mean KOOS symptoms started from 53.1 to 79 points, mean KOOS sports started from 38.7 to 74.8 points, mean KOOS quality of life started from 43.3 to 73.7 points. The mean IKDC score improved by 27.7 ± 15.7 points. All these scores have been significantly improved (*p* < 0.05). MRI assessments confirmed the successful integration of repair tissue with a mean MOCART 2.0 score of 80.5 ± 12.5 points.

**Conclusion:**

The minced autograft cartilage technique with PRP provides favourable early clinical and radiological outcomes for limited chondral defects in the knee. This method offers a single‐procedure approach with minimal grafting requirements and does not necessitate a laboratory or specialized personnel, unlike other techniques.

**Level of Evidence:**

Level III retrospective multicentric study.

**Abbreviations**
ACL: anterior cruciate ligament
BMI: body mass index
CID: clinically important difference
ICRS: International Cartilage Repair Society
IKDC: International Knee Documentation Committee
KOOS: Knee Injury and Osteoarthritis Outcome Score
MFX: microfracture
MOCART: Magnetic Resonance Observation of Cartilage Repair Tissue
MRI: magnetic resonance imaging
NAS: Numeric Analogue Scale
PF: patellofemoral
PRP: platelet‐rich plasma
QOL: quality of life
SKV: Self Knee Value

## INTRODUCTION

Orthopaedic surgeons frequently encounter injuries to knee articular cartilage, with an estimated 200,000–300,000 procedures for cartilage defects performed annually in the United States [5]. Their aetiology is often traumatic in a young population. There are frequently associated lesions, which are predominantly medial meniscus injuries and anterior cruciate ligament (ACL) ruptures [10, 32]. Surgical interventions, however, provide options to treat cartilage defects. In response to this type of condition, Cugat et al. have published a one‐stage surgical technique involving a cartilage autograft enriched with growth factors [9]. These preliminary reports were case reports and short case series so the promising outcomes need to be confirmed in larger series [7, 8]. Additionally, numerous studies confirm the effectiveness of platelet‐rich plasma (PRP) in early clinical outcomes addressing cartilage defects [11, 28]. Animal studies have also shown the benefits of autologous chondral PRP matrix implantation [23].

This study aims to assess if patients with knee cartilage defects who received one‐stage surgery with minced autograft cartilage and PRP showed clinical improvement after at least one year of follow‐up. The condition of the articular cartilage and the repair tissue was assessed using magnetic resonance imaging (MRI) and the Magnetic Resonance Observation of Cartilage Repair Tissue (MOCART 2.0) scoring system.

The hypothesis of this work was that favourable clinical and radiological outcomes could be achieved at short‐term follow‐up using one‐stage minced cartilage and PRP implantation to treat limited cartilage defects.

## MATERIALS AND METHODS

After review board approval, a multicentric, non‐randomized retrospective analysis was performed from a prospectively collected database in two sports medicine centres. All patients gave valid consent to participate in this study (CPP no. 2023‐A00910035).

**Figure 1:**
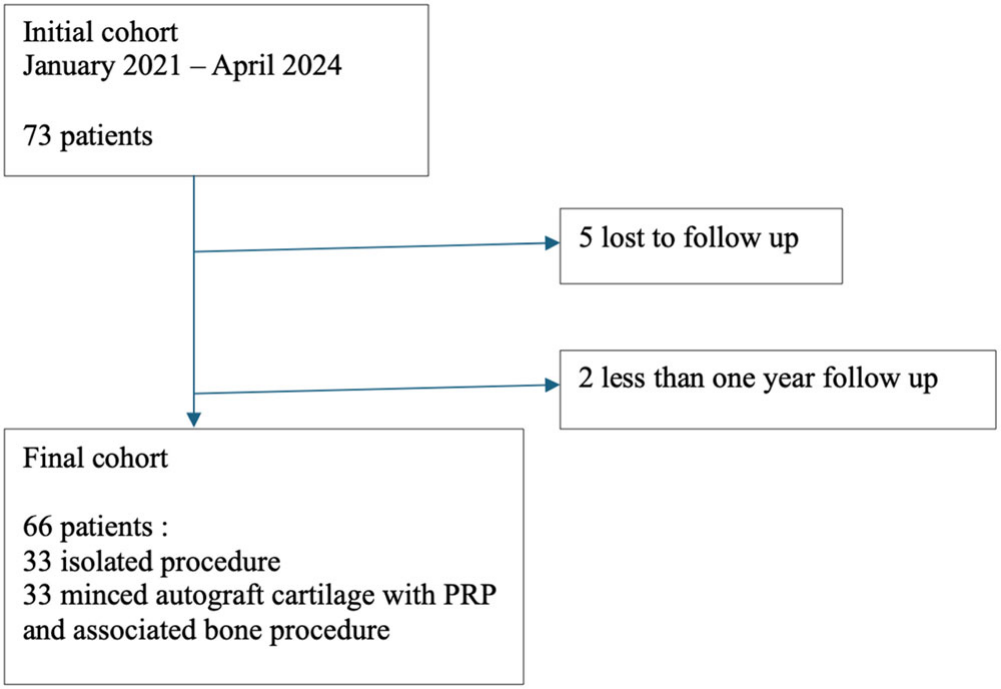
Flowcharts.

All patients between 18 and 50 years of age who underwent the minced cartilage autograft and PRP procedure between January 2021 and April 2023 for knee cartilage defect and fulfilled the included criteria were analyzed (Figure 1). Inclusion criteria included symptomatic International Cartilage Repair Society (ICRS) Grade III/IV chondral defects [4] and not improved patients despite well‐conducted medical treatment such as infiltrations and analgesics (*n* = 66). Exclusion criteria included a follow‐up of less than 1 year (*n* = 5) and knee osteoarthritis Ahlbäck grade <2. Seventy‐three patients met the inclusion criteria, and 5 patients were excluded, 66 patients were finally included in this study. The surgery was performed by two trained senior surgeons specializing in knee surgery and practising in sports surgery centres.

Patient data were extracted from their medical records. Information on sex, body mass index (BMI), the location of the cartilage defect and its size were collected. Any previous history of surgery on the affected joint was documented. Thirty‐three patients underwent the procedure alone and a further 33 had associated bone procedures.

Associated procedures included ACL ligamentoplasty, medial patella‐femoral ligament ligamentoplasty, tibial valgization osteotomy and tibial tubercle transfer.

### Preoperative assessment

All patients underwent comprehensive medical histories and physical examinations of the knee. Several evaluation scores were collected during pre‐operative consultations, including the Knee Injury and Osteoarthritis Outcome Score (KOOS), International Knee Documentation Committee (IKDC) score and Self Knee Value (SKV). A thorough clinical assessment of the knee was performed to determine if the patient was suitable for any associated procedures. MRI was used to assess the size and location of the chondral defects, as well as any concomitant pathologies, such as meniscal tears or ligamentous insufficiencies.

### Surgical Technique

Surgical intervention was performed either arthroscopically or via open surgery, based on the surgeon's preference. Thirty‐two patients had arthroscopic surgery and 34 had open surgery. This approach facilitated the assessment of the defect size and location. The AUTOCART™ (Arthrex®) procedure was employed.

A small amount of cartilage was harvested from a lesser weight‐bearing area of the knee—either the medial or lateral condylar ridge, or the trochlear notch—or directly adjacent to the defect. A blood sample was drawn from the patient and centrifuged for five minutes. The resultant serum was then enriched with growth factors. Half of the obtained PRP was mixed with the harvested cartilage sample, and the other half was combined with the Thrombinator™ (Arthrex®) system. The prepared cartilage paste was applied to the defect and subsequently covered with thrombin, all within a dry environment.

### Post‐operative rehabilitation

When the cartilage defect was located in the patellofemoral (PF) joint, the leg was maintained in extension using an external brace for the first 48 h post‐surgery. This was subsequently replaced with an articulated brace, permitting knee flexion of 0–30° during the initial 2 weeks. Flexion was then increased to 0–60° for the subsequent 2 weeks, and further to 0–90° until the end of the sixth week. Weight‐bearing was restricted to a light 15 kg for the initial 6 weeks. Starting from the sixth week, a progressive increase in weight‐bearing by 20 kg per week was implemented, and activities such as cycling and swimming were allowed. The patient could resume low‐impact sports starting from the third month post‐operatively, and high‐impact sports were permitted from the sixth month onwards.

When the defect was located in the femorotibial joint, the leg was immobilized in extension for 48 h, after which it was allowed unrestricted movement. The re‐weighting protocol followed was identical to that described for the PF joint.

### Clinical assessment

Patients were assigned subjective functional scores both pre‐operatively and at the last follow‐up. The KOOS evaluates pain, symptoms, ability to perform activities of daily living (ADLs), sports and recreation functions, and knee‐related quality of life (QOL) [28]. The International Knee Documentation Committee (IKDC) [17] and the SKV, which assigns a score between 0 and 100, were also utilized.

### MRI assessment

MRI was systematically performed at the last follow‐up for all patients, excluding those with any metal equipment, such as osteotomy plates, which could cause artefacts. Imaging was conducted using a 1.5 T scanner. Standard proton density and T2‐weighted fat‐saturated images were acquired in both coronal and sagittal planes (slice thickness: 3 mm; field of view: 15 cm; 512 matrices in at least one axis for proton density images, and a minimum of 256 matrices in one axis for T2‐weighted images). Additionally, axial proton density fat‐saturated images were obtained (slice thickness: 3–4 mm; field of view: 14–15 cm; minimum 224 matrices in at least one axis) [13].

The parameters of graft repair are closely aligned with the components of the Magnetic Resonance Observation of Cartilage Repair Tissue (MOCART 2.0) scoring system. These components include signal intensity, graft infill, border integration, surface contour, structure, subchondral lamina, subchondral bone and effusion [14, 18, 19]. Additionally, this scoring system is noted for its high interobserver reliability and has demonstrated a correlation with clinical outcomes [3]. The MOCART 2.0 score was made by two independent orthopaedic surgeons not involved in the patient's treatment.

### Statistical analysis

All statistical analyses were performed using Microsoft Excel version 16.75. and XLSTAT 2023.2.0. The normality was tested with the Shapiro–Wilk test. If the normality was verified, the difference between groups was assessed using a Student's *t* test, with an alpha risk set to 5% (*α* = 0.05) and a beta risk set to 20% (*β* = 0.20). If the normality was not verified, the difference between groups was assessed using the Wilcoxon signed‐rank test. The alpha risk was set to 5% (*α* = 0.05), and the Beta risk was set to 20% (*β* = 0.20). *p* Values ≤0.05 were considered significant. Correlations were performed using Pearson and Spearman correlation coefficients.

## RESULTS

The study population was predominantly male, comprising 52 men (79%), with an average age of 30.4 years (standard deviation [SD] = 11.2) and an average BMI of 23.8 (SD = 3.5). The cartilage defect was located in the femoral‐patellar compartment in 46% and in both the medial and lateral femoral‐tibial compartments in 27% each (Table 1).

**Table 1:** Baseline characteristics.

	*n*	%
Sex		
Male	52	79
Female	14	21
Location		
Femoral‐patellar	30	46
Internal	18	27
External	18	27
Stage ICRS		
III	52	79
IV	14	21
Harvested site		
received site	59	89
femoral notch	6	9
trochlea	1	2
Procedure		
Isolated	33	50
Bone procedure associated	33	50
Open surgery	34	52
Arthroscopy	32	48
	Mean	SD
Age (years)	30.4	11.2
BMI (kg/m^2^)	23.8	3.5
Surface (cm^2^)	2.6	1.3

Abbreviations: ICRS, International Cartilage Repair Society; BMI, body mass index; SD, standard deviation.

The mean size of the defect was 2.6 cm^2^ (SD = 1.3). Most defects were classified as Stage III (*n* = 52, 79%) according to the ICRS classification.

**Figure 2:**
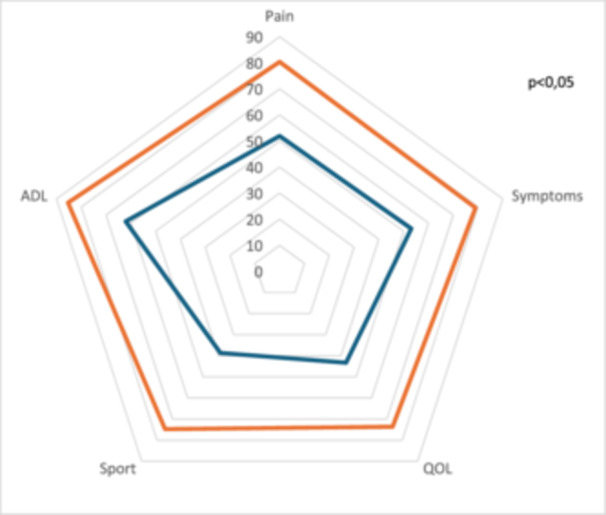
KOOS before surgery (blue) versus last follow‐up (orange). KOOS, Knee Injury and Osteoarthritis Outcome Score.

The mean follow‐up was 20.5 months (min 12; max 36.8). The mean KOOS total improvement was 26.2 points (SD = 12.6, *p* < 0.05), starting from 45.3 to 71.5 points. The greatest improvement concerned the KOOS sports, with 32.8 points (SD = 20.1, *p* < 0.05), starting from 38.7 to 74.8 points. Mean KOOS pain started from 51.9 to 80.4 points, mean KOOS activities of daily (ADLs) living started from 62.1 to 85.3 points, mean KOOS symptoms started from 53.1 to 79 points, mean KOOS quality of life (QOL) started from 43.3 to 73.7 points (Figure 2). There was also a significant improvement in the IKDC from 43.9 to 73.7 and the SKV from 35.2 to 81.2 score (*p* < 0.05).

The MOCART 2.0 score could be realized on 46 patients (70%) at the last follow‐up. The mean score was 80.5 (SD = 12.5).

Moderate correlations were found between delta IKDC and preoperative IKDC (0.63), as well as delta SKV and preoperative IKDC (0.66).

No significant differences were observed based on lesion localization and surface lesion groups.

**Figure 3:**
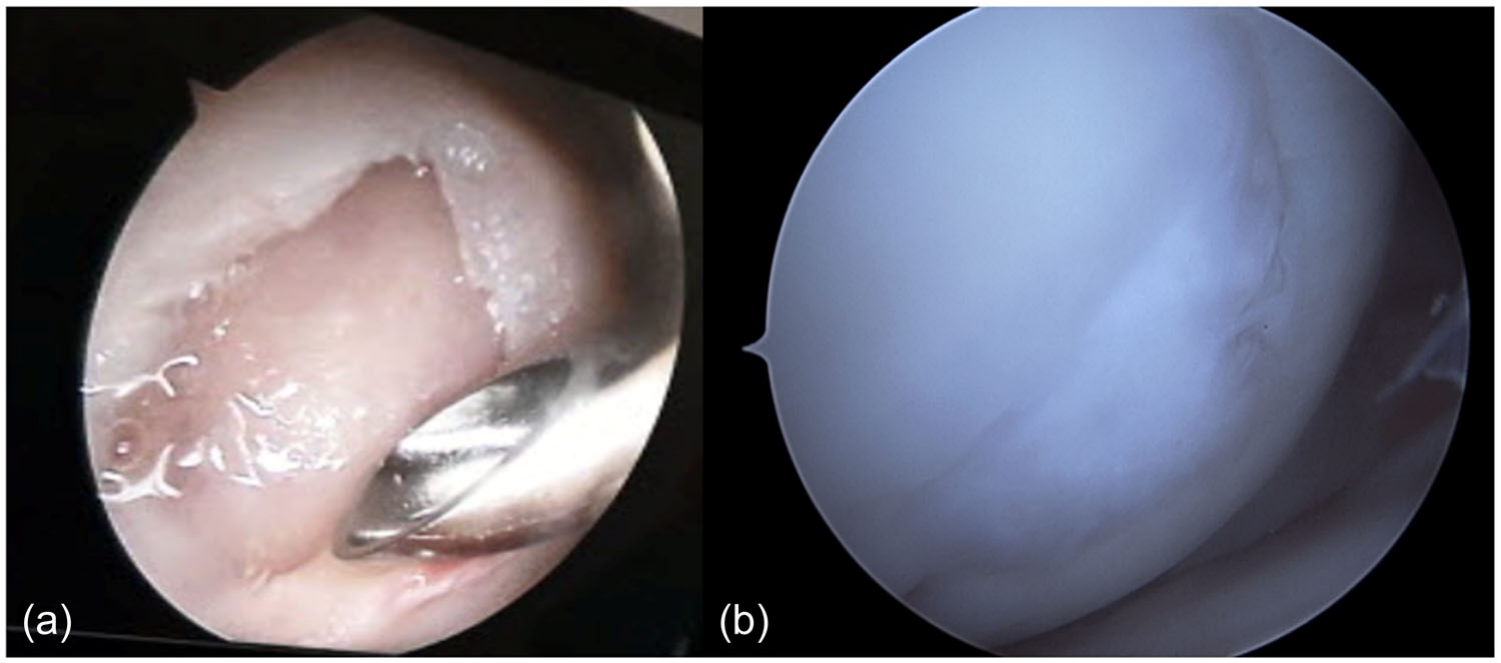
(a) Cartilage defect in lateral condyle. (b) Same defect 18 months after procedure.

Two patients required repeat surgery. One for an early site infection: the follow‐up protocol was straightforward, involving surgical debridement and appropriate antibiotic therapy for a duration of 6 weeks. The other patient had a repeat surgery due to discomfort at 18 months of follow‐up caused by fibrin glue that had become lodged in the notch, resulting in cyclops syndrome (Figure 3). However, nothing explaining the symptoms was found during revision surgery. No further complications were reported.

## DISCUSSION

The main findings of the current study support the hypothesis that favourable early clinical outcomes are achieved, as evidenced by improvements in the KOOS, IKDC and SKV scores. Additionally, radiological assessments reveal positive outcomes, with satisfactory MOCART 2.0 scores observed at the final follow‐up. According to Chahal et al. the findings of the present study appear to be clinically significant by reaching the clinically important difference (CID) for all the KOOS parameters [6]. The CID for patient‐reported outcomes after knee cartilage repair is 8.3 for KOOS Pain, 8.8 for KOOS ADL, 30.0 for KOOS Sports and Recreation, 18.8 for KOOS QOL and 9.2 for IKDC.

This finding could be explained by biological phenomena. Harvesting healthy chondrocytes and applying them in a ‘bare’ zone with a favourable environment will promote their outgrowth [17, 27]. This way, the production of matrix will be effective. It is interesting to note that the harvested cartilage from the defect edge is vital [26] and shows superior redifferentiation of chondrocytes compared to non‐weight‐bearing cartilage [2, 15]. A healthy joint status leads to higher chances of success for cell‐based techniques [29].

The use of small autologous cartilage pieces to treat osteochondral lesions was first demonstrated by Albrecht [1] in an experimental rabbit model. The literature includes numerous series, including several single‐stage cartilage autografts which minimize surgical risks associated with a two‐step procedure. Massen et al. [20] carried out a prospective study of 27 patients receiving mince cartilage grafts, they found a significant improvement in the Numeric Analogue Scale (NAS) involving pain and function (*p* < 0.01) at 2 years of follow‐up. In this study, the graft was fixed with fibrin glue in the femorotibial compartment and with Chondro Gide membrane® (Geistlich Pharma) in the PF. Wodzig et al. [33] also published radiological outcomes of cartilage autograft on 18 patients; the mean MOCART 2.0 score was 65 ± 18.9 which is less than in the present study (81.4). According to their results, the lateral condyle had better radiological outcomes than the medial condyle. Słynarski et al. [31] took an interest in cartilage autograft and bone marrow mononucleated cells seeded into a polyactive scaffold in a single‐step procedure. The results of the prospective study on 40 patients demonstrated a significant KOOS improvement of 21 points. The IKDC also increased significantly. Two patients underwent a surgical re‐intervention due to graft hypertrophy. Siebold et al. [30] studied 30 patients at 3 years of follow‐up after another one‐step procedure, which consisted of autologous chondrocyte implantation (ACI) implantation using chondrospheres®. 86.6% of patients were satisfied with the surgery and all the KOOS fields were improved significantly. The mean MOCART score at 3 years of follow‐up was 60 ± 21. These clinical findings are consistent with this early outcome study but the minced autograft with PRP technique seems to offer a better improvement of radiological outcome according to the MOCART 2.0 score.

Other studies investigated the implantation of autologous chondrocytes in a single procedure with a longer follow‐up. Runer et al. [25] analyzed 34 patients with ACI fixed by chondrogide® or fibrin glue at the 60‐month follow‐up. The mean IKDC score was 71.6 ± 14.8 and 14.2% of surgery‐related complications were related directly to minced cartilage. Niemeyer et al. [22] studied the first ACI generation on 70 patients, the survey of the graft was 71.4% at 10 years, the mean IKDC was 74 ± 17.3 and the mean MOCART score was 44.9 ± 23.6.

All these studies concur that the implantation of autologous chondrocytes appears to be a good and safe option in the surgical treatment of pre‐arthritic osteochondral defects at midterm follow‐up.

Some recent studies compare cartilage grafts to other surgical therapeutic options. A systematic review of Kraeutler et al. [16] found that patients undergoing microfracture (MFX) or first/third‐generation ACI for articular cartilage lesions in the knee can be expected to experience improvement in clinical outcomes at midterm to long‐term follow‐up without any significant difference between the groups. Mundi et al. [21] published another systematic review and meta‐analysis comparing bone marrow stimulation, ACI and OAT, they found no significant difference between this treatments in improving function and pain at midterm follow‐up. A more recent systematic review by Dhillon et al. [12], which takes into account only third‐generation of ACI versus MFX demonstrated that at short‐term follow‐up, third‐generation ACI had lower failure rate and greater improvement in patient‐reported outcomes compared with MFX for focal chondral defects. The meta‐analysis published by Riboh et al. [24] suggested that there is no difference between MFX and ACL at 2 years of follow‐up about functional results and repeat surgery, but from 5 to 10 years of follow‐up, the ACI technique would reduce the re‐operation risk and provide higher quality‐repair tissue. The results of meta‐analyses are not unequivocal. This heterogeneity in results is partly explained by the existence of several generations of ACI. Nevertheless, according to the meta‐analyses by Dillon and Riboh et al., it appears that the more recent ACI techniques offer better outcomes.

This study has several limitations. First, it focuses on short‐term outcomes. It is therefore not possible to conclude on the protective effect against the risk of osteoarthritis. Second, there is no comparative group treated with alternative osteochondral procedures such as ACI, Osteochondral Autograft Transfer System, or MTX Technique. Third, a significant portion of the cartilage defects were located at the femoral condyle, which may limit the generalizability of the findings to other areas of the knee joint. Additionally, it should be noted that the overall outcomes might be less favourable in patients with cartilage lesions of the patella compared to those with other locations [14]. Fourthly, the studied technique was combined with other bony procedures in a significant number of patients, complicating the extrapolation of results.

The mean defect size in this study was 2.6 cm^2^ (SD = 1.3), which is small; these, findings cannot be extrapolated to large defects, especially those greater than 4 cm^2^.

To confirm the efficacy of the minced autograft cartilage and PRP technique, further prospective studies involving larger sample sizes and extended follow‐up periods are necessary.

This finding showed good clinical and radiological outcomes of the studied procedure for small cartilage defects. Compared to other techniques, the results seem to be coherent clinically but with a better radiological improvement. The specific advantage of this technique is a single procedure performed in the operating room without the involvement of a laboratory or specialized personnel, which is an advantage compared to other ACI techniques, and a minimal grafting intake to avoid complications due to harvesting, which makes an advantage over mosaic plastic techniques.

## CONCLUSION

The minced autograft cartilage technique with PRP provides good early clinical and radiological outcomes for limited chondral defects in the knee.

## AUTHOR CONTRIBUTIONS

Arthur Barbaret collected the data and wrote the manuscript. Frank Wein collected the data and reviewed the manuscript. Christophe Jacquet reviewed the manuscript. Matthieu Ollivier reviewed the manuscript and made statistics analysis.

## CONFLICT OF INTEREST STATEMENT

Frank Wein is Arthrex® consultant. Matthieu Ollivier is Newclip® consultant. The remaining authors declare no conflicts of interest.

## ETHICS STATEMENT

This study has been approved by the local ethics committee and authorized by the French personal data protection committee (Comité de protection des personnes) under no. 2023‐A00910035. Patient consent was expressed orally.

## Data Availability

Data were collected from patients' computerized medical records and during consultations. Their consent was required for data collection.
